# LAR Downregulation Protects the Astrocytic U251 and Cocultured SH-SY5Y Cells in a Rotenone-Induced Parkinson’s Disease Cell Model

**DOI:** 10.3390/ijms241311111

**Published:** 2023-07-05

**Authors:** Wei Zheng, Xiao Han, Bing Han, Gang Li, Jing Gan, Tian Wang, Bo Xu, Jie He, Wenxiao Du, Xiaolin Cao, Zhenhua Wang

**Affiliations:** 1 Center for Mitochondria and Healthy Aging, College of Life Sciences, Yantai University, Yantai 264005, China; weizheng@ytu.edu.cn (W.Z.);; 2 School of Pharmacy, Yantai University, Yantai 264005, China; 3 College of Life Sciences, Yantai University, Yantai 264005, China

**Keywords:** LAR, Parkinson’s disease, astrocyte, mitochondrial function, neuron, rotenone

## Abstract

Leukocyte common antigen-related protein tyrosine phosphatase (LAR) is a member of the protein tyrosine phosphatase family that serves as a key regulator of cellular survival. It is also involved in neurodevelopment and brain disorders. This study was designed to investigate the role of LAR in a cell-based model of Parkinson’s disease (PD) in which U251 and SH-SY5Y cells were used as models of astrocytes and dopaminergic neurons, respectively. Cell viability, cell death, cell morphology, protein phosphorylation and expression, ATP levels, reactive oxygen species (ROS) generation, and mitochondrial membrane potential were analyzed in the wild-type (WT) and heterozygous LAR-knockout astrocytoma U251 cells to assess the cell state, signal transduction, and mitochondrial function. LAR downregulation showed a protective effect in rotenone-exposed U251 cells by increasing cell viability, reducing cell mortality, and restoring appropriate cellular morphology. LAR downregulation enhanced IGF-1R phosphorylation and downstream signal transduction as evidenced by increases in the Akt and GSK-3β phosphorylation, as well as the upregulation of NRF2 and HO-1. The downregulation of LAR also augmented DJ-1 levels in these cells. The enhanced Akt and GSK-3β phosphorylation contributed to a reduced Bax/Bcl2 ratio and suppressed apoptosis after rotenone exposure. Heterozygous LAR-knockout U251 cells exhibited higher mitochondrial function evidenced by increased mitochondrial membrane potential, ATP contents, and reduced ROS production compared to the WT cells following rotenone exposure. Further studies showed that the astrocytic protection mediated by the heterozygous knockout of LAR was associated with the activation of Akt. A specific Akt inhibitor, MK2206, reduced the cell viability, Akt and GSK3β phosphorylation, and HO-1 and NRF2 expression in U251 cells exposed to rotenone. Astrocytes provide structural and metabolic support to maintain neuronal health. Astrocytic glial cell-derived neurotrophic factor (GDNF) production is vital for dopaminergic neuron survival. Heterozygous LAR-knockout U251 cells produced higher amounts of GDNF than the WT cells. The SH-SY5Y cells cocultured with heterozygous LAR-knockout U251 cells exhibited greater viability than that of cells cocultured with WT U251 cells in response to rotenone. Together, these findings demonstrate that the heterozygous knockout of LAR in astrocytes can play a key role in protecting both astrocytic cells and cocultured neurons in a rotenone-induced cell-based model of PD. This neuroprotective effect is attributable to the augmentation of IGF1R-Akt-GDNF signaling and the maintenance of astrocytic mitochondrial function.

## 1. Introduction

Parkinson’s disease (PD) is a common neurodegenerative disease that causes severe movement disorders [1], with over 8.5 million PD patients throughout the world as of 2019 [2]. To date, no curative treatments for PD have been developed [2]. Degeneration of dopaminergic neurons in the substantia nigra pars compacta and dopamine loss in the striatum are the most prominent features of PD [3]. These impairments lead to tremors, postural instability, and bradykinesia [3]. Current evidence suggests that PD is caused by a combination of several factors, including environmental toxins, genetic mutations, and aging [4]. The main pathological mechanisms underlying PD include α-synuclein accumulation, mitochondrial dysfunction, and oxidative stress in neuronal cells [5]. In addition, non-neuronal cells including astrocytes also play important roles in the progression of PD [5,6,7].

Astrocytes, the most abundant glial cell type in the central nervous system (CNS), play a crucial role in sustaining neuron health [5]. Increasing evidence suggests that astrocyte dysfunction and death also lead to dopaminergic neuron degeneration in PD [8,9,10]. Astrocytes not only provide structural support to neurons but also regulate extracellular ion balance in the central nervous system [8] and transport glutamine to neurons [11]. They are important components for the maintenance of the blood–brain barrier, which is disrupted in PD patients [11]. During neuroinflammation, astrocytes surround the inflammatory location and create a barrier between the inflammatory tissue and healthy cells, thus playing a neuroprotective role [11]. Antioxidants and neurotrophic factors produced by astrocytes are essential for dopaminergic neuron development and survival [12,13]. α-Synuclein aggregation within dopaminergic neurons is a hallmark of PD that leads to Lewy body formation in neurons, disrupts normal cellular function, and results in neuron cell death [5]. α-Synuclein aggregates can be released by neurons into the extracellular space via exocytosis, whereupon they can be engulfed and degraded by the astrocytes [14,15,16]. As such, astrocytes protect dopaminergic neurons by clearing the excess harmful α-synuclein [5]. Mitochondrial dysfunction is another major mediator of PD development and progression [17]. Worn-out mitochondria are usually removed through mitophagy to maintain neuronal health [18,19]. Astrocytes are also responsible for the clearance of these damaged mitochondria from neurons [20]. Recent work has shown that induced pluripotent stem cell-derived astrocytes from humans can also donate healthy mitochondria to dopaminergic neurons and rescue them from neurotoxicity [21].

Rotenone is a natural compound that can be extracted from the seeds and roots of several leguminous and liana plants and is used as a broad-spectrum insecticide [22]. As rotenone can induce typical PD characteristics, including Lewy body formation in neurons of the substantia nigra and neurodegeneration, rotenone-exposed animal and cell models are frequently used as experimental tools for studies of PD [23]. Rotenone causes mitochondrial malfunction, excessive production of reactive oxygen species (ROS), and neuronal death by inhibiting mitochondrial complex I and inducing α-synuclein aggregation [24,25,26]. Mitochondria also regulate astrocyte functions, mitochondrial dysfunction in astrocytes causes imbalanced glutamate metabolism, neuronal excitotoxicity, and ROS overproduction [7,27,28]. These factors are contributors to the pathogenesis of PD [7].

Leukocyte common antigen-related protein tyrosine phosphatase (LAR) is a member of the protein tyrosine phosphatase (PTP) family and serves as a key regulator of phosphorylation signal transduction and downstream physiological effects including cell survival, proliferation, and differentiation [29]. Prior work has shown that LAR plays a role in neurodevelopment and brain disorders [29]. LAR suppresses the phosphorylation of receptor tyrosine kinases including ephrin type-A receptor 2 (EphA2), epidermal growth factor receptor (EGFR), and insulin-like growth factor-1 receptor (IGF-1R) and, therefore, reduces downstream signaling activity mediated through pathways including the PI3K-Akt pathway [30,31,32]. PI3K-Akt signaling promotes cell survival and reduces apoptosis in the CNS [33]. Akt is also among the most important apoptosis-inhibiting proteins [33]. A member of the PTP family, PTP1B, was recently shown to play an important role in PD [34].The aim of this study was to explore the role of LAR in PD using a rotenone-induced cell model. U251 and SH-SY5Y cells were employed as models for astrocytes and dopaminergic neurons, respectively.

## 2. Results

### 2.1. LAR Expression Is Reduced in D1 and D2 Cells Compared with WT U251 Cells

**Figure 1:**
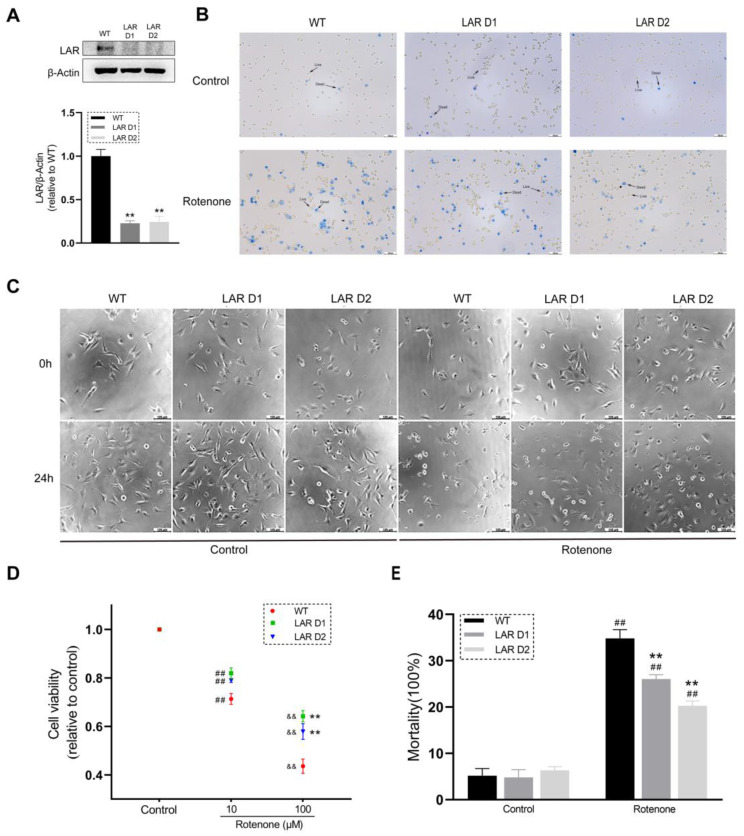
Heterozygous LAR knockout protects astrocytic U251 cells from rotenone toxicity. (**A**) Representative Western blotting results for LAR in WT and heterozygous LAR knockout D1 and D2 U251 cells with corresponding quantification normalized to β-actin. (**B**) Images of trypan blue stained WT, D1, and D2 U251 cells treated with or without rotenone. Scale bar = 100 μm. (**C**) Cell morphology images of WT, D1, and D2 U251 cells treated with or without 100 μM rotenone. Scale bar = 100 μm. (**D**) Scatter plot quantification analysis of the viability of the U251 cell lines after being exposed to rotenone. (**E**) Quantification of U251 cell mortality after being exposed to rotenone. Data are the means ± SEM from triplicate experiments. ## *p* < 0.01 vs. control, ** *p* < 0.01 vs. WT cells, && *p* < 0.01 vs. 10 μM rotenone group.

Western blot experiments were used to confirm LAR levels in the U251 cell lines. As expected, the LAR levels in the heterozygous LAR-knockout D1 and D2 cells were markedly reduced and were at approximately 20% of the levels observed in WT cells (Figure 1A).

### 2.2. Heterozygous LAR Knockout Increases U251 Cell Viability and Maintains Normal Cell Morphology in the Presence of Rotenone

The cell viability and morphology in cells treated with or without rotenone were examined to study the impact of LAR on astrocyte survival in conditions of rotenone toxicity. Rotenone significantly reduced the viability of the U251 cell lines. The D1 and D2 cells exhibited relatively higher viability than WT cells following exposure to a 100 μM rotenone concentration (Figure 1D), which also altered the morphology of these cell lines. The D1 and D2 cells exhibited more normal morphological characteristics compared with WT cells following rotenone treatment (Figure 1C).

### 2.3. Reductions in LAR Expression Reduce Cell Mortality following Rotenone Exposure

Exposure to a 100 μM rotenone dose led to higher mortality in U251 cells, whereas a significantly reduced mortality was evident for the D1 and D2 cells (Figure 1B,E). As a result, a 100 μM rotenone dose was used for the following experiments.

### 2.4. Rotenone Induces Greater Increases in DJ-1 Expression and Akt Phosphorylation in D1 and D2 Cells

**Figure 2:**
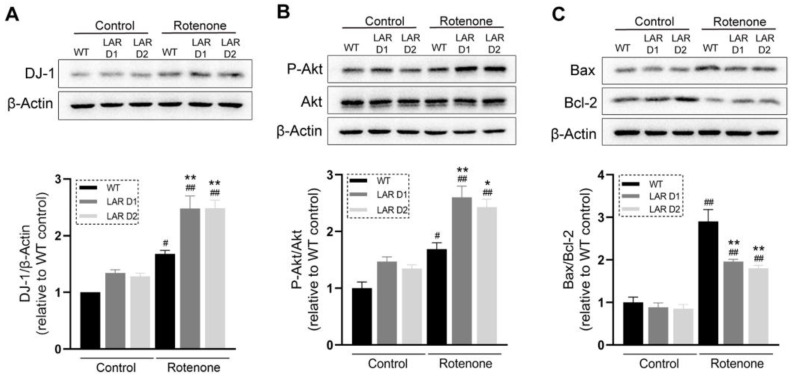
Heterozygous LAR knockout modulates Akt phosphorylation and DJ-1, Bax, and Bcl-2 protein levels in U251 cells. (**A**) Representative Western blotting results for DJ-1 and β-actin and corresponding quantification, with β-actin used for normalization. (**B**) Representative Western blotting results for P-Akt, Akt, and β-actin and corresponding quantification, with Akt used to normalize P-Akt levels. (**C**) Representative Western blotting results for Bax, Bcl-2, and β-actin and corresponding quantification, with data indicating the Bax levels relative to Bcl-2 levels in these samples. Data are the means ± SEM from triplicate experiments. # *p* < 0.05, ## *p* < 0.01 vs. control, * *p* < 0.05, ** *p* < 0.01 vs. WT.

To explore the mechanistic basis of the higher cell viability and lower cell mortality observed for the heterozygous LAR-knockout cells after rotenone exposure, Akt phosphorylation and protein deglycase-1 (DJ-1) protein levels were measured. The DJ-1 levels in the U251 cell lines increased upon rotenone exposure, and D1 and D2 cells expressed significantly higher level of DJ-1 relative to WT cells (Figure 2A). Akt phosphorylation was also enhanced in these U251 cell lines after rotenone challenge, with these increases being markedly higher in the D1 and D2 cells relative to WT cells in the presence of rotenone (Figure 2B). The DJ-1 and P-Akt levels of D1 and D2 seemed to be slightly higher than those of the WT in the absence of rotenone, but these differences were not statistically significant (Figure 2A,B).

### 2.5. Rotenone Induces Greater Increases in the Bax/Bcl-2 Ratio in WT U251 Cells

The Bax/Bcl-2 ratio increased in these U251 cell lines after rotenone exposure, with WT cells manifesting a higher Bax/Bcl-2 ratio than the LAR heterozygous knockout cell lines (Figure 2C).

### 2.6. Akt Inhibition Reduces the Viability of Rotenone-Treated U251 Cell Lines

**Figure 3:**
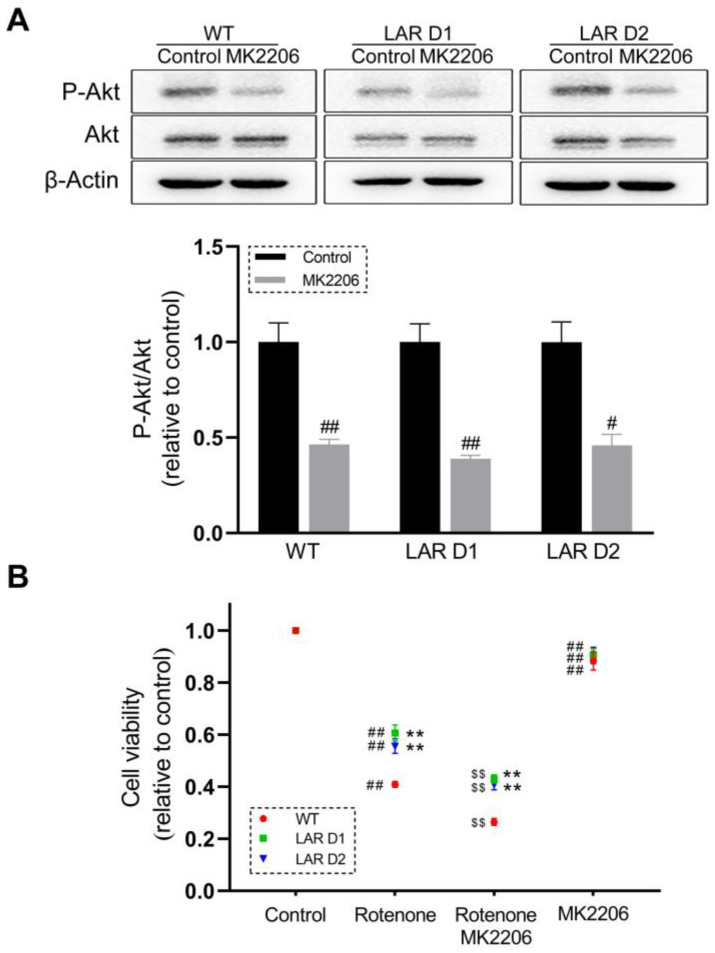
Akt inhibition further suppresses the viability of the U251 cell lines in the presence of rotenone. (**A**) Representative Western blotting results for Akt, P-Akt, and β-actin levels, with corresponding quantification in which P-Akt levels were normalized to total Akt levels. (**B**) Scatter plot quantification of the viability of U251 cell lines after being exposed to rotenone and MK2206. Data are the means ± SEM from triplicate experiments. # *p* < 0.05, ## *p* < 0.01 vs. control, ** *p* < 0.01 vs. WT, $$ *p* < 0.01 vs. rotenone group.

The Akt-specific inhibitor MK2206 was used to test whether Akt signaling is involved in the observed differences in viability between WT and D1 or D2 cells. MK2206 reduced Akt phosphorylation by ~50% in these U251 cell lines (Figure 3A). Cotreatment with MK2206 and rotenone additionally suppressed the viability of all cell lines when compared with rotenone exposure alone (Figure 3B).

### 2.7. Rotenone Induces Greater Increases in GSK-3β and Akt Phosphorylation Levels in D1 and D2 Cells That Are Reversed by MK2206 Treatment

**Figure 4:**
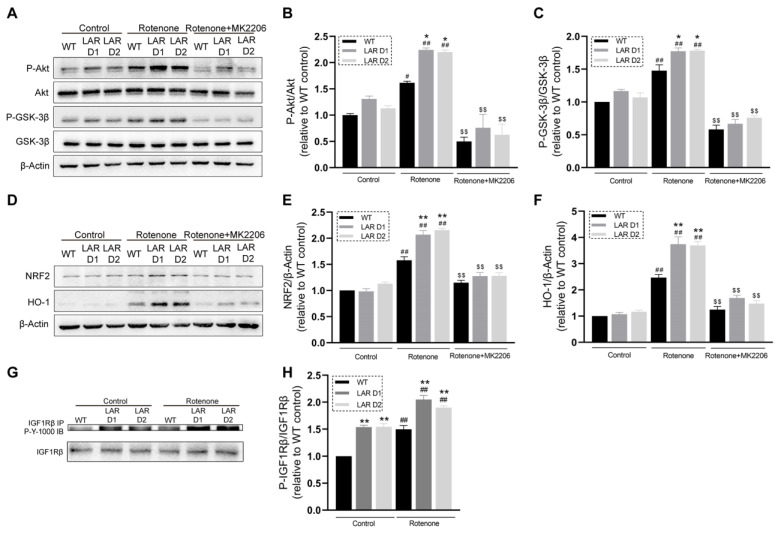
Heterozygous LAR knockout potentiates IGF-1R-Akt signaling, increased GSK-3β phosphorylation and promoted NRF2 and HO-1 expression in the presence of rotenone, while Akt inhibition suppressed these changes. (**A**) Representative Western blotting results for P-Akt, Akt, P-GSK-3β, GSK-3β, and β-actin. (**B**) The quantification of P-Akt levels which were normalized to total Akt. (**C**) The quantification of P-GSK-3β levels were normalized to total GSK-3β. (**D**) Representative Western blotting results for NRF2, HO-1, and β-actin. (**E**) The quantification of NRF2 levels were normalized to β-actin. (**F**) The quantification of HO-1 levels were normalized to β-actin. (**G**) Representative Western blotting results for P-IGF-1R and IGF-1R. (**H**) The quantification of P-IGF-1R levels were normalized to IGF-1R. Data are the means ± SEM from triplicate experiments. # *p* < 0.05, ## *p* < 0.01 vs. control, * *p* < 0.05, ** *p* < 0.01 vs. WT, $$ *p* < 0.01 vs. rotenone group.

The phosphorylation of Akt and the downstream signaling protein glycogen synthase kinase 3β (GSK-3β) were next analyzed in the absence or presence of rotenone and MK2206. Rotenone increased GSK-3β and Akt phosphorylation in the U251 cell lines. These phosphorylation increases were significantly greater in the D1 and D2 cells compared with the WT cells. Cotreatment with rotenone and MK2206 reduced the observed GSK-3β and Akt phosphorylation levels compared with rotenone treatment alone (Figure 4A–C).

### 2.8. Rotenone Induces Greater Increases in HO-1 and NRF2 Protein Levels in D1 and D2 Cells That Are Reversed by MK2206 Treatment

In response to rotenone exposure, these U251 cell lines showed elevated levels of nuclear factor erythroid 2-related factor 2 (NRF2) and heme oxygenase-1 (HO-1). In D1 and D2 cells, these increases were significantly greater than in the WT cells. Cotreatment with rotenone and MK2206 significantly reduced the NRF2 and HO-1 levels compared with rotenone exposure alone (Figure 4D–F).

### 2.9. Heterozygous LAR Knockout Enhances Rotenone-Induced IGF-1Rβ Phosphorylation

An immunoprecipitation approach was used to assess IGF-1R phosphorylation, revealing that rotenone exposure promoted IGF-1R phosphorylation in the U251 cells. The IGF-1R phosphorylation in D1 and D2 cells was significantly enhanced relative to the WT cells (Figure 4G,H).

### 2.10. Heterozygous LAR Knockout Suppresses Rotenone-Induced ROS Production, While Cotreatment with Rotenone and MK2206 Induces More ROS Production than Rotenone Alone

**Figure 5:**
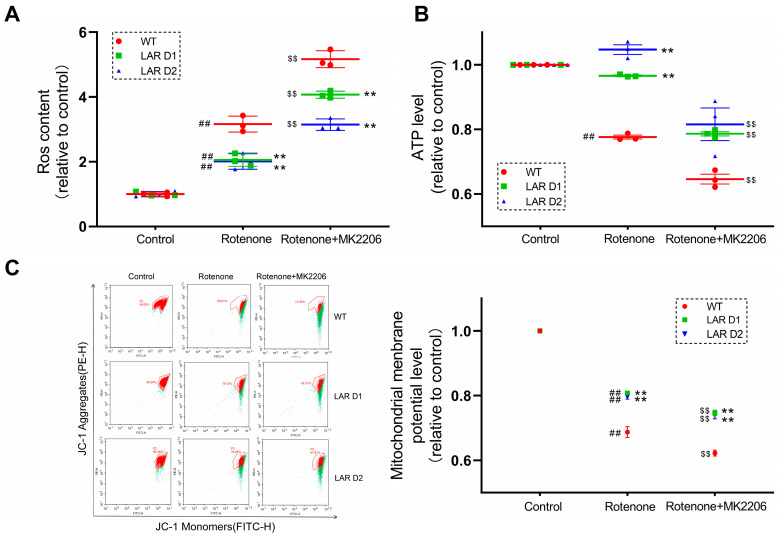
Heterozygous LAR knockout reduces ROS production and promotes mitochondrial function under conditions of rotenone exposure, while Akt inhibition further increases ROS production and suppresses mitochondrial function under conditions of rotenone treatment. (**A**) Quantitative analysis of the ROS production by U251 cell lines treated with or without rotenone and MK2206. (**B**) Quantitative analysis of ATP levels in U251 cells treated with or without rotenone and MK2206. (**C**) JC-1 fluorescent signal as measured via flow cytometry with corresponding quantification of the mitochondrial membrane potential levels in U251 cell lines treated with or without rotenone and MK2206. Red dots represent the JC-1 fluorescent signal in the untreated cells, green dots represent the JC-1 signal change after rotenone and MK2206 exposure. Data are the means ± SEM from triplicate experiments. ## *p* < 0.01 vs. control, ** *p* < 0.01 vs. WT, $$ *p* < 0.01 vs. rotenone alone.

Next, ROS production was measured, revealing that rotenone exposure led to ROS overproduction in these U251 cell lines. In comparison to the D1 and D2 cells, ROS levels were noticeably higher in WT cells (Figure 5A). Cotreatment with both rotenone and MK2206 led to further increases in ROS production compared with rotenone exposure alone (Figure 5A). WT cells also had considerably greater ROS levels compared to D1 and D2 cells cotreated with rotenone and MK2206 (Figure 5A).

### 2.11. Heterozygous LAR Knockout Enhances Mitochondrial Membrane Potential (ΔΨm) under Rotenone Exposure and Cotreatment with Rotenone and MK2206

Next, mitochondrial membrane potential was measured in these cells, revealing that rotenone exposure led to a reduction in ΔΨm in the U251 cell lines. Notably, ΔΨm was substantially lower in the WT cells than in the D1 and D2 cells. Cotreatment with rotenone and MK2206 further reduced ΔΨm values in these U251 cells, and the ΔΨm values in the WT cells remained significantly lower than those in the D1 and D2 cells (Figure 5C).

### 2.12. Heterozygous LAR Knockout Cells Exhibit Higher ATP Levels than WT Cells after Rotenone Exposure and Cotreatment with Rotenone and MK2206

An analysis of the ATP levels showed that rotenone treatment decreased ATP levels in the WT cells, with the ATP levels in these WT cells being considerably lower compared to the ATP levels in the D1 and D2 cells after rotenone exposure. Cotreatment with rotenone and MK2206 additionally decreased ATP levels in these U251 cell lines (Figure 5B).

### 2.13. Astrocytic Heterozygous LAR Knockout Contributes to Enhanced GDNF Production and Exerts Stronger Neuroprotective Effects on SH-SY5Y Cells

**Figure 6:**
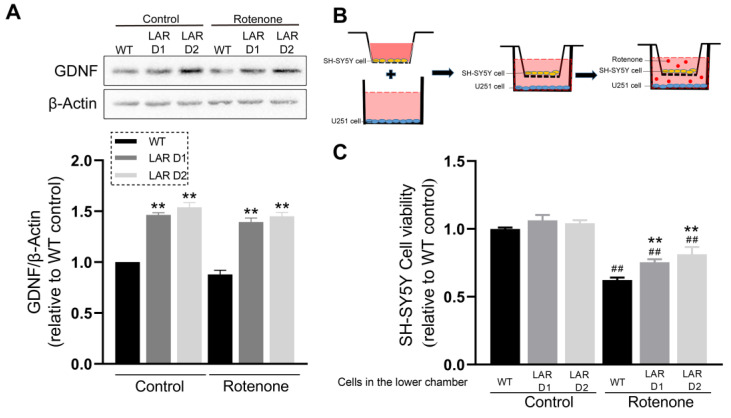
Heterozygous LAR knockout potentiates astrocytic GDNF production and neuroprotective activity. (**A**) Representative Western blotting results for GDNF and β-actin with corresponding quantification in which β-actin was used for normalization. (**B**) Schematic diagram showing the set-up used for the cell coculture assay. (**C**) Quantitative analysis of SH-SY5Y cell viability following coculture with the indicated U251 lines treated with or without rotenone. Data are the means ± SEM from triplicate experiments. ## *p* < 0.01 vs. control, ** *p* < 0.01 vs. WT.

The GDNF levels were measured, and they were shown to be considerably elevated in D1 and D2 cells compared to WT cells in both the presence and absence of rotenone (Figure 6A). The SH-SY5Y cells were cocultured with the U251 cell lines either with or without rotenone to assess how LAR affects the neuroprotective properties of these astrocytes (Figure 6B). In comparison to SH-SY5Y cells cocultured with WT U251 cells, the viability of SH-SY5Y cells cocultured with D1 or D2 U251 cells after rotenone exposure was significantly improved (Figure 6C).

## 3. Discussion

The CNS consumes high amounts of glucose and oxygen and engages in continuous metabolic activity to satisfy its own energy demands [35]. This characteristic leads to the production of a large volume of ROS [35]. In the neurons of the substantia nigra, the metabolism of cytosolic free dopamine causes additional ROS generation, which makes this area of the brain especially vulnerable to oxidative stress [36]. This is a major contributor to neurodegeneration in PD [37]. Astrocytes surrounding the neurons play key roles in antioxidant production, ROS detoxification, and neuroprotection [38]. However, when the amount of ROS exceeds the detoxifying capacity of these astrocytes, ROS can cause mitochondrial dysregulation, additional ROS production, ΔΨm reduction, and cytochrome c release into the cytoplasm [39,40]. These processes, consequently, cause the activation of caspases, astrocyte apoptosis, and the loss of their neuroprotective function [39,40].

Previous studies have shown that the rotenone treatment of rat C6 astrocytoma cells reduces mitochondrial activity, increases ROS production, and decreases the viability of these cells [41]. Here, rotenone exposure markedly reduced the viability and increased the mortality of the astrocytic U251 cells. Heterozygous LAR knockout rescued these cells from rotenone toxicity. Morphological analyses revealed that rotenone altered cell morphology, while decreased LAR expression partially restored the normal morphology of these cells. Rotenone exposure also induced the overproduction of ROS and ΔΨm decreases in these astrocytic cells, while the heterozygous knockout of LAR reversed these effects. Rotenone additionally lowered the levels of ATP in WT cells without any comparable effect in LAR heterozygous knockout cells. These findings suggest that heterozygous LAR knockout protects astrocytic mitochondrial function and thus shields these cells from mitochondria-mediated cell death.

**Figure 7:**
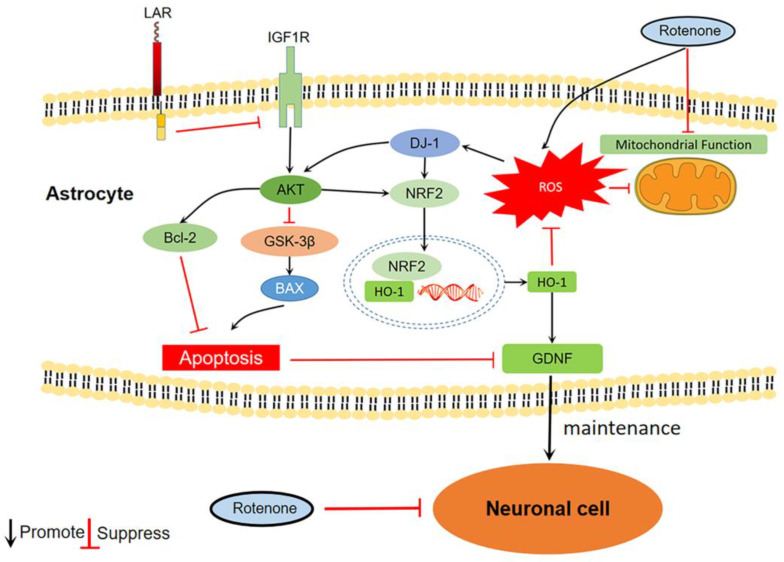
A schematic overview of the molecular mechanisms underlying LAR-mediated activity.

In order to explore the molecular mechanisms underlying the astrocytic-protective function of heterozygous LAR knockout, the phosphorylation of Akt and the DJ-1, Bcl-2, and Bax protein levels in these cells were measured. DJ-1 is primarily expressed in astrocytes and is upregulated under conditions of oxidative stress, playing important roles in neuroprotection, antioxidant activity, and mitochondrial function [5]. It activates Akt by suppressing PTEN [42,43,44]. Akt is crucial for cell survival, and its disruption is common in PD [45,46]. Selective loss of dopaminergic neurons frequently coincides with decreased Ser473 Akt phosphorylation within the brain in the context of PD [47]. The activation of Akt contributes to the upregulation of Bcl-2 and inhibition of Bax [48,49]. Oligomers formed by translocated Bax on the mitochondrial outer membrane cause pores in the membrane that allows for the release of cytochrome c and subsequent cell death [39]. Bcl-2 binds to Bax and prevents this Bax oligomerization, thereby inhibiting consequent pore formation [50]. The ratio between Bax and Bcl-2 is usually used as an indicator of apoptosis [50]. In the present study, rotenone exposure increased DJ-1 expression in U251 cells, which may be due to oxidative stress. DJ-1 levels in the LAR-downregulated cells were significantly higher than that of WT after rotenone exposure. Akt, being downstream of DJ-1, displayed a similar phosphorylation pattern. Rotenone enhanced Akt phosphorylation, while heterozygous LAR knockout further increased Akt phosphorylation in the presence of rotenone. The Bax/Bcl-2 ratio was increased in rotenone-exposed cells, while LAR-downregulated D1 and D2 cells exhibited a lower Bax/Bcl-2 ratio compared with the WT after rotenone exposure. These results suggest that rotenone exposure increases DJ-1 protein expression in turn enhancing Akt phosphorylation. These increases were further promoted by the heterozygous knockout of LAR. The increased levels of Akt phosphorylation observed in the LAR-downregulated cells contributed to a reduction in the Bax/Bcl-2 ratio, thereby protecting against rotenone-induced apoptosis (Figure 7). However, the mechanistic cause for the increase in the DJ-1 levels in the heterozygous LAR knockout cells under rotenone treatment still needs to be further studied.

As Akt phosphorylation was prominently enhanced in the LAR-downregulated cells, the Akt-specific inhibitor MK2206 was used to test whether this activation of Akt signaling pathway was responsible for the higher viability of the heterozygous LAR knockout cells. MK2206 inhibited Akt in these U251 cell lines as expected. The U251 cell lines viability was significantly decreased when cotreated with rotenone and MK2206. This shows that increased Akt phosphorylation is a significant factor in the increased cell viability reported in LAR-downregulated cell lines.

Glycogen synthase kinase 3β (GSK-3β) is an activator of neuronal apoptosis [51]. It phosphorylates Bax and promotes its translocation to the mitochondria and consequent cell death [51]. As a direct substrate, GSK-3β is inhibited by Akt through phosphorylation at Ser9 [52]. NRF2 is a master transcriptional regulator of cellular anti-oxidative response. It is constantly degraded by the proteasome because it interacts with the Kelch-like ECH-associated protein 1 (KEAP1) under normal circumstances [53]. Oxidative stress changes the structure of KEAP1 and prevents NRF2 degradation. NRF2 can then be translocated to the nucleus and binds to antioxidant-response element (ARE) sequences in the chromosomal DNA [53]. Many antioxidant proteins, such as HO-1, are expressed as a result of this process [53]. NRF2 also upregulates key antioxidant mediators including superoxide dismutase (SOD) and glutathione (GSH) [54]. Astrocytic NRF2 activation plays a major role in neuroprotection [55]. Importantly, Akt assists NRF2 activation by inhibiting its interaction with KEAP1, and DJ-1 upregulates NRF2 expression [56]. The GSK-3β and Akt phosphorylation, along with the protein HO-1 and NRF2 levels, were determined in untreated cells, rotenone-exposed cells, and cells cotreated with rotenone and MK2206 to gain insight into the molecular mechanism involved in the LAR-related astrocytic protection against rotenone toxicity. The HO-1 and NRF2 protein levels were increased in response to rotenone, along with GSK-3β and Akt phosphorylation. Cotreatment of cells with rotenone and MK2206 inhibited GSK-3β and Akt phosphorylation and expression of HO-1 and NRF2 compared to only rotenone treatment. Cotreatment with MK2206 and rotenone also additionally increased ROS production and reduced ΔΨm and ATP levels compared with rotenone alone. This indicates that higher levels of Akt phosphorylation in the heterozygous LAR-knockout cells contribute to the enhanced phosphorylation and inactivation of GSK-3β, thus suppressing the Bax/Bcl-2 ratio and apoptotic cell death. In heterozygous LAR-knockout cells, hyper-phosphorylated Akt together with upregulated DJ-1 promote the expression of NRF2 which triggers the production of a range of intracellular antioxidants. This action significantly reduces the ROS levels in these cells, preserves normal mitochondrial function, and suppresses cell death (Figure 7). Akt thus plays a central role in these signaling pathways.

To determine why Akt signaling is markedly stronger in the heterozygous LAR-knockout cells, a search for upstream proteins was conducted, revealing that IGF-1R phosphorylation was significantly upregulated in the heterozygous LAR-knockout cells. IGF-1 and its corresponding cell surface transmembrane receptor IGF-1R are essential regulators of multiple cell functions including survival, proliferation, antioxidant activity, and neuroprotection [57,58]. Ligand binding to the IGF-1R extracellular α-subunit induces a conformational change for this receptor, which then leads to the autophosphorylation of the tyrosine residues of the IGF1R transmembrane β-subunit [59]. As a result, the PI3K-Akt pathway and various subsequent signaling cascades are activated [57,58]. LAR directly dephosphorylates IGF-1R and thus abrogates downstream cell proliferation and migration [30]. In the present study, rotenone exposure increased IGF-1R phosphorylation in the U251 cells, while the reduction of LAR expression promoted IGF-1R phosphorylation. This indicates that LAR suppresses Akt signaling by dephosphorylating and suppressing IGF-1R in astrocytes. When LAR protein levels are reduced, IGF-1R is hyper-phosphorylated, contributing to the hyper-activation of Akt and its downstream signaling pathway (Figure 7). Intracellular ROS can cause the oxidation of active-site cysteines present in protein tyrosine phosphatases (PTPs), thereby reducing their activity [60]. Since some other PTPs also dephosphorylate IGF-1R [61], the elevated levels of IGF-1R phosphorylation observed under conditions of rotenone exposure may be due to the partial inhibition of these PTPs by ROS overproduction.

Astrocytes are essential mediators of neuroprotection that function by producing GDNF, which is important for dopaminergic neuron survival [8]. To investigate the effect of LAR heterozygous knockout on astrocytic GDNF production and its neuroprotective capability in this rotenone-induced PD model system, the cocultures of wild-type (WT) U251 or LAR heterozygous knockout U251 cells with SH-SY5Y cells were treated with or without rotenone. Astrocytic GDNF production was found to be significantly higher when LAR was heterozygously knocked out. Since HO-1 overexpression in substantia nigra upregulates GDNF production in glial cells [62], the higher GDNF level in LAR heterozygous knockout cells may be induced by the higher HO-1 and NRF2 levels. Moreover, the SH-SY5Y cells cocultured with U251 cells were less likely to survive after being exposed to rotenone. Under rotenone-inducing conditions, the SH-SY5Y cells cocultured with the LAR heterozygous knockout U251 cells showed markedly increased vitality compared to SH-SY5Y cells cocultured with WT U251 cells. This suggests that LAR heterozygous knockout astrocytic cells possess stronger neuroprotective capabilities in the context of PD due to their greater viability and more robust GDNF production (Figure 7).

## 4. Materials and Methods

### 4.1. Cell Culture

Human U251 glioblastoma cells were purchased from the Cell Bank of the Chinese Academy of Sciences (Shanghai, China). The heterozygous LAR-knockout cell lines LAR D1 and LAR D2 are two different cell monoclones generated by UBIGENE, Guangzhou, China with the wild-type U251 cells (WT) using a CRISPR/cas9 technique. These cells were cultured using DMEM (Gibco, Waltham, MA, USA), supplemented with 10% fetal bovine serum (FBS, TIANHANG, Huzhou, Zhejiang, China) and penicillin/streptomycin (Gibco, Waltham, MA, USA) in a 37 °C 5% CO_2_ incubator.

### 4.2. Cell Viability and Morphology Analyses

An MTT assay was used to measure cell viability. Briefly, the U251 WT, LAR D1, and LAR D2 cell lines were exposed to 10 or 100 μM rotenone (Sigma-Aldrich, St. Louis, MO, USA) for 24 or 72 h with or without 100 nM MK2206 (Absin, Shanghai, China) at 37 °C. The cells on a 96-well plate were then treated with 0.5 mg/mL MTT. After 3 h, the growth media was discarded, and 150 μL of DMSO was added to each well before the plates were oscillated for 15 min to distribute the solution. SpectraMax Paradigm Multi-Mode Microplate Reader (Molecular Devices, San Jose, CA, USA) absorbance values at 595 nm were determined for each well. After 24 h of treatment with 100 μM rotenone, the cell morphology was analyzed by taking images of the WT, LAR D1, and LAR D2 cells under a microscope (Leica Microsystems CMS GmbH, Wetzlar, Germany).

### 4.3. Cell Mortality Assay

The cell mortality was assessed via trypan blue staining. The U251 WT, LAR D1, and LAR D2 cell lines cultured in 6-well plates were treated with 100 μM rotenone for 24 h. The cells were then removed from each well using PBS, centrifuged at 1500× *g* for 1 min, and resuspended in 1 mL of PBS. Next, 100 μL of 0.08% Trypan Blue was added to each 100 μL of cell suspension, and the resulting mixtures were incubated for 3 min at room temperature. The cells were subsequently counted using a cell counter (Denovix, Wilmington, DE, USA). Approximately 3 × 500 cells were assessed for Trypan Blue staining for each sample. Cell mortality was calculated as 100% × number of Trypan Blue stained cells/total cell number. In addition, the stained cell suspension was mixed evenly before depositing a droplet onto a microscope slide for imaging with a microscope (Leica Microsystems CMS GmbH, Wetzlar, Germany).

### 4.4. Western Blotting and Protein Immunoprecipitation

U251 cell lines were exposed to 100 μM rotenone and 100 nM MK2206 for 90 min or 24 h for analyses of phosphorylation and protein levels, respectively. After treatment, cells were placed on ice and rinsed with pre-cooled PBS twice. Lysis buffer (150 mmol/L NaCl, 100 mg/mL PMSF, 1% NP-40, 50 mmol/L Tris–HCl (pH 7.4), 0.1% SDS) was then added to these cells prior to incubation on ice for 15 min. Collected cells were centrifuged (15 min, 13,000× *g*, 4 °C), after which supernatants were collected. Protein levels therein were detected with an enhanced BCA protein detection kit (Beyotime Biotechnology, Shanghai, China). For immunoprecipitation experiments, IGF-1R was precipitated from cell lysate samples containing an equal amount of protein using anti-IGF-1Rβ (Cell Signaling Technology, Danvers, MA, USA) followed by incubation with protein A agarose beads (Absin, Shanghai, China). Samples were separated via 5%, 6%, 10%, 12%, or 15% gels SDS-PAGE (Mini-PROTEAN, Bio-Rad, Hercules, CA, USA) based on protein size. Separated proteins were transferred onto Immunobilon-P PVDF membranes (Millipore, Burlington, MA, USA), which were subsequently blocked using 5% BSA (Sigma-Aldrich, St. Louis, MO, USA) in TBS-T solution (10 mmol/L Tris-base, 68 mmol/L NaCl, pH 7.5, 0.1% Tween 20) for 1 h at room temperature. After incubating in the suggested concentration of primary antibody overnight at 4 °C, membranes were washed thoroughly with TBS-T, and incubated in the corresponding peroxidase-conjugated secondary antibody for 1 h followed by an additional round of washing. Proteins were visualized with a 5200 Multi Luminescent image analyzer (Tanon Science and Technology, Shanghai, China), and Image Proplus 6.0 was used for quantification. The following antibodies were used in this study (all from Cell Signaling Technology unless otherwise indicated): LAR, IGF-1Rβ, Akt, Phospho-Akt Ser473, GSK-3β, Phospho-GSK-3β Ser9, Bax, Bcl-2, DJ-1, NRF2, HO-1, Phospho-Tyrosine PY1000, and GDNF (Abcam, Cambridge, UK) (Table 1). Horseradish peroxidase (HRP) Goat anti-Rabbit immunoglobulin G (IgG) was used as the secondary antibody.

### 4.5. ROS Analyses

Cells were incubated with rotenone and MK2206 for 6 h before they were washed three times with pre-warmed DMEM. Then, 10 μM of DCFH-DA fluorescent probe was added to the cells. The probe was removed after 30 min incubation at 37 °C. The cells were then collected and centrifuged at 900× *g* for 5 min. The cell pellet was resuspended with PBS, the fluorescent signal from DCFH-DA bound to the cell was analyzed using an ACEA Novocyte flow cytometer (ACEA Biosciences, San Diego, CA, USA) and Novo Express software (version 1.6.1).

### 4.6. Measurement of Mitochondrial Membrane Potential (ΔΨm)

Following incubation with rotenone and MK2206 for 24 h, cells were washed three times using pre-warmed DMEM followed by incubation with 5 µg/mL of the fluorescent probe JC-1 (Thermo Fisher Scientific, Waltham, MA, USA) for 30 min at 37 °C. The cells were washed again with pre-warmed PBS and digested with trypsin. The collected cells were centrifuged (5 min, 900× *g*) and resuspended using PBS, after which the fluorescent JC-1 signal was detected with an ACEA Novocyte flow cytometer (ACEA Biosciences, San Diego, CA, USA) and the Novo Express software (version 1.6.1). The ratio of red to green fluorescence was analyzed to calculate the mitochondrial membrane potential (ΔΨm).

### 4.7. ATP Assay

Cellular ATP content was analyzed with a luciferase-based luminescence-enhanced ATP assay kit (Beyotime, Shanghai, China). Following rotenone treatment for 24 h, cells were rinsed using chilled PBS and lysed with chilled ATP-releasing buffer. Following lysate centrifugation (5 min, 12,000× *g*, 4 °C), supernatants were collected and combined with an ATP testing working solution. ATP levels in these samples were then measured with a plate reader as above (Molecular Devices, Sacramento, CA, USA).

### 4.8. Cell Coculture

Transwell Polyester membrane chambers (Corning, New York, NY, USA) were used for U251-SH-SY5Y cell coculture. Cell culture medium was added to the upper and lower chambers and incubated overnight at 37 °C. SH-SY5Y cells were seeded in the upper chambers, while the U251 WT, LAR D1, or LAR D2 cell lines were seeded in the lower chambers. After 24 h, a normal culture medium or medium containing rotenone was added to the upper and lower chambers (Figure 6B). After 72 h, 0.5 mg/mL MTT was added to each upper chamber and incubated for 3 h, after which this medium was replaced with 200 μL of DMSO. This solution was then transferred into a 96-well plate, and the absorbance at 595 nm was analyzed using a plate reader as above (Molecular Devices, San Jose, CA, USA).

### 4.9. Statistical Analyses

Data are the means ± SEM. The analyses were carried out with GraphPad Prism 8.0. All data were analyzed via one-way ANOVAs with Tukey’s post hoc test. *p* < 0.05 was the cut-off for statistical significance.

## 5. Conclusions

In conclusion, this study found that heterozygous LAR-knockout grants astrocytic cells a higher degree of viability, enhanced GDNF production, and stronger neuroprotective capacity in a cell-based model of PD. The mechanistic basis for these effects is associated with the activation of IGF-1R and Akt. Akt activation leads to the Bax/Bcl-2 ratio reduction and cell apoptosis suppression. Akt and DJ-1 further drives the upregulation of NRF2 and HO-1, consequently suppressing ROS production, preserving mitochondrial function, and increasing GDNF production. These higher levels of astrocytic viability and GDNF production also contributed to the augmented viability of cocultured neuronal SH-SY5Y cells in a rotenone-induced PD model system. Thus, inhibiting LAR to modulate the viability and function of astrocytes offers a novel therapeutic strategy for PD as LAR performs a vital role in the neuroprotective function of astrocytes.

## Figures and Tables

**Table 1:** The primary antibodies used.

Antibody	Catalogue Number	Animal	Dilution
Akt	4060	Rabbit	1:2000
Bax	41162	Rabbit	1:1000
Bcl-2	15071	Mouse	1:1000
DJ-1	5933	Rabbit	1:1000
GDNF	Ab176564	Rabbit	1:2000
GSK-3β	12456	Rabbit	1:1000
HO-1	26416	Rabbit	1:1000
IGF-1Rβ	9750	Rabbit	1:1000
LAR	61611	Rabbit	1:1000
NRF2	12721	Rabbit	1:1000
Phospho-Akt Ser473	4060	Rabbit	1:2000
Phospho-GSK-3β Ser9	9323	Rabbit	1:1000
PY1000	8954	Rabbit	1:2000

## Data Availability

Not applicable.
